# Dietary Features Are Associated with Differences in the Urinary Microbiome in Clinically Healthy Adult Dogs

**DOI:** 10.3390/vetsci11070286

**Published:** 2024-06-27

**Authors:** Emily L. Coffey, Zoe W. Becker, Andres M. Gomez, Aaron C. Ericsson, Julie A. Churchill, Erin N. Burton, Jennifer L. Granick, Jody P. Lulich, Eva Furrow

**Affiliations:** 1 Department of Veterinary Clinical Sciences, College of Veterinary Medicine, University of Minnesota, Saint Paul, MN 55108, USA; beck0957@umn.edu (Z.W.B.); churc002@umn.edu (J.A.C.); grani003@umn.edu (J.L.G.); lulic001@umn.edu (J.P.L.); furro004@umn.edu (E.F.); 2 Department of Animal Science, College of Food, Agricultural, and Natural Resource Sciences, University of Minnesota, Saint Paul, MN 55108, USA; gomeza@umn.edu; 3 Department of Veterinary Pathobiology, College of Veterinary Medicine, University of Missouri, Columbia, MO 65211, USA; ericssona@missouri.edu; 4 Department of Veterinary and Biomedical Sciences, College of Veterinary Medicine, University of Minnesota, Saint Paul, MN 55108, USA; burt0233@umn.edu

**Keywords:** urinary microbiome, urobiome, canine, dog, dietary diversity, nutrition

## Abstract

**Simple Summary:**

The microbiome refers to the vast community of microorganisms such as bacteria that inhabit a particular host or ecosystem. Recently, it was discovered that the urine from healthy dogs hosts its own unique microbiome, known as the urobiome. These microbial organisms play important roles in maintaining urinary system health and preventing disease. Although the gut microbiome is heavily influenced by diet, it remains unknown how nutritional features impact the urobiome. Therefore, the purpose of this work was to determine how dietary features alter the urobiome’s composition in clinically healthy dogs. Specifically, we examined how nutrient intake (protein, fat, and crude fiber), commercial diet brands, and dietary diversity (i.e., the number of unique food sources consumed each day) altered the abundance and diversity of bacteria present in the canine urobiome. We discovered that both commercial diet brands and dietary diversity were associated with distinct shifts in the composition of the urobiome. This discovery highlights the complex relationships between diet and urinary microbes, and these findings could ultimately lead to novel dietary strategies to promote urobiome health.

**Abstract:**

Nutrition plays an important role in shaping the gut microbiome composition, although the impact of diet on the urinary microbiome (i.e., urobiome) remains unknown. The aim of this pilot study was to discover how nutritional features affect the diversity and composition of the urobiome in dogs. Dietary histories were obtained for 15 clinically healthy adult dogs, including limited nutrient (protein, fat, crude fiber), commercial diet brand, and dietary diversity profiles. The urine samples were collected via cystocentesis, followed by sequencing of the bacterial 16S rRNA gene. The data were analyzed to determine associations between major nutrients and dietary sources with the urobiome’s composition. The protein, fat, and crude fiber contents had no statistically significant effect on the alpha or beta diversity. However, the beta diversity values differed (PERMANOVA; *p* = 0.017, R^2^ = 0.10) between dogs fed one commercial diet brand compared to dogs consuming any other brand. The beta diversity values also differed (*p* = 0.019, R^2^ = 0.10) between dogs consuming more diverse daily diets compared to those consuming less diverse diets (≥3 or <3 unique food sources, respectively). Overall, the results of this pilot study suggest that diet might impact the urobiome and support further exploration of the relationship between diet and the urobiome’s composition in dogs.

## 1. Introduction

The previous dogma maintained that urine, in the absence of infection, is sterile. However, emerging research shows that even urine from healthy individuals contains diverse populations of bacteria, collectively known as the urobiome [1,2,3,4,5,6]. These microbial populations play essential roles in maintaining urinary tract health, and alterations within the urobiome are linked to several urogenital diseases [6,7,8,9,10,11]. Delineating the dynamics of host–microbe interactions within the urinary tract is necessary to understand how specific features of the urobiome promote or protect against disease. Ultimately, such information could be harnessed to manipulate the urobiome as a novel disease management tool for a variety of disorders. The diet is readily modifiable and an attractive target for strategically altering the urinary microenvironment to support resident bacteria, yet the impact of nutrition on the urobiome’s composition remains unknown.

Defining the relationship between nutritional features and the urobiome is an essential first step in leveraging diet to produce targeted effects on the urobiome. Diet is one of the largest contributors shaping the gut microbiome [12,13], and in theory has the potential to produce both direct and indirect effects on urinary microbial communities. Urobiome colonization is not well understood, although growing evidence supports the presence of a gut–bladder axis [14,15]. For instance, the gut is a known reservoir for uropathogens that cause urinary tract infections [16,17]. Urobiome colonization could occur secondary to perineal contamination by gut microbes, which subsequently ascend the urinary tract. Thus, diet-induced shifts in the gut microbiome could alter the urobiome through direct organism transmission. Furthermore, many metabolites produced from microbial interactions with dietary substrates have systemic effects on the host, including the urinary tract. For example, short-chain fatty acids produced from the microbial breakdown of dietary fibers can reduce inflammatory responses in the kidneys [18], which could have indirect effects on urinary microbes. Finally, dietary features exert downstream effects on the urinary microenvironment, such as the urine pH and urinary metabolomic profiles [19,20,21], which could subsequently affect urobiome communities. In a study of patients with type 2 diabetes mellitus, specific dietary factors altered the relationships between urinary microbes and urinary interleukin-8 levels, suggesting that dietary strategies could be used to augment bladder inflammation [22]. Despite these potential mechanisms, the currently available research is focused largely on relationships between diet and the gut microbiome, with growing data to support a gut–urinary microbiome axis [12,13,14,15]. Minimal data are available that directly investigate if or how dietary features impact the urobiome’s composition.

Evaluating the impact of diet on the human urobiome raises specific challenges, particularly given the high degree of daily variation in human food consumption. Dogs typically consume more consistent diets and also represent an emerging animal model for urobiome research. Like humans, dogs harbor a diverse urobiome and suffer from several of the same urogenital disorders that have been linked to human urobiome alterations [23,24,25,26,27]. The dominant taxa comprising the urobiomes of dogs and humans are similar [27], and parallel taxonomic shifts have been observed between dogs and humans in certain disease states, such as urinary stone disease [23]. Furthermore, the gut microbiome in dogs displays more similarities in taxonomic and microbial genomic profiles to that of humans than do those of mice or pigs, and dogs demonstrate similar shifts in gut microbial populations in response to diet as those observed in humans [28]. Thus, dogs represent a powerful translational model for studying the relationships between nutritional features and urobiome composition.

The aim of this pilot study was to characterize the impact of nutritional features on urobiome composition in healthy dogs, including limited nutrient profiles, commercial diet brand, and dietary diversity. We hypothesized that nutritional profiles are associated with differences in urobiome diversity and composition. The characterization of the relationship between diet and urobiome composition in dogs could expose novel strategies for augmenting urobiome health through diet and nutrition.

## 2. Materials and Methods

### 2.1. Animals

The study participants were recruited through an email announcement to the University of Minnesota’s College of Veterinary Medicine employees and students in May 2021 for urobiome research [29]. This study did not involve human participants. All clients provided written, informed consent for their dog’s participation, with the study protocol approved by the University of Minnesota’s Institutional Animal Care and Use Committee (protocol number 2005-38140A) [29]. Only spayed or neutered dogs between the ages of 1 and 7 years were eligible. The exclusion criteria were antimicrobial or immunosuppressive therapies within 3 months of enrollment; a history or clinical signs of lower urinary tract disease (hematuria, stranguria, pollakiuria, or dysuria); active dermatologic disease or visible dermatologic lesions in the perivulvar, preputial, or inguinal areas; a history or clinical signs of recent (within 3 weeks of the study) gastrointestinal disease (vomiting, diarrhea, hematochezia, borborygmus, hyporexia, anorexia, or abdominal bloating); or a change in the primary diet within one month prior to study participation. The signalment, body weight, body condition score (BCS, 1–9 scale), medical history, and dietary history data were recorded for each participant.

### 2.2. Diet History Collection

The current diet histories were collected directly from each owner using a thorough dietary history questionnaire that included questions about the brand, specific product name, and amount fed per day of any food or treats consumed by the study participant. Details about the source (i.e., commercial brand and specific product name) and nutritional content were recorded for each dog’s primary diet and for any reported treats. The primary diet was defined as the diet comprising at least 60% of the dog’s daily caloric intake. For commercially available diets and treats, the guaranteed analyses for each food or treat source were used to collect the nutrient data. This information was collected directly from the manufacturer and product guides. For dogs that consumed human foods as treats or as a component of the primary diet, the nutritional information was collected from the U.S. Department of Agriculture Nutrient Database (https://fdc.nal.usda.gov, accessed on 1 July 2022) [30]. The nutrient features recorded for all dogs included diet ingredient lists and caloric information (kcal/kg of food, kcal/cup or can of food, and kcal from each dietary source as a percent total of daily intake). Additionally, the amounts of protein, fat, and crude fiber (recorded as g/100 kcal) consumed daily by each dog were recorded. The nutrients for each dog were calculated by adding the respective nutrient content (protein, fat, crude fiber) from each daily food source, relative to the percent of daily caloric intake for that food source. Two clients reported that their dogs consumed treats from a commercial service that delivers mixed treat sources (BarkBox™, New York, NY, USA). The specific treats from this source given prior to urine collection were not always known, although the number of treats given daily was reported. Thus, the maximum daily calories possibly consumed via treats were calculated using the most caloric treats available in the delivery. This ensured that the primary diet still comprised >60% of daily calories, even if only the highest calorie treats were consumed. The downstream statistical analyses for these two dogs used the average nutrient values from all treats that the dogs might have consumed during the month prior to urine collection.

The dogs were assigned into one of two diet groups based on the commercial brand of the primary diet. Diet group 1 included a subset of dogs that were all consuming diets of the same commercial brand (Purina^®^, Nestlé Purina Petcare, St. Louis, MO, USA), while diet group 2 included dogs consuming any other commercial brand (Royal Canin^®^ USA Inc., Mars Inc., Saint Charles, MO, USA; NutriSource^®^ Pet Foods, Perham, MN, USA; Fromm^®^ Family Pet Food, Mequon, WI, USA). The dogs were also assigned to one of two dietary diversity groups based on the overall number of dietary and treat sources [31]. The groups were designated as low dietary diversity (LDD) if fed < 3 dietary and treat sources and high dietary diversity (HDD) if fed ≥ 3 sources. Both dogs consuming treats from the commercial delivery service were designated as HDD. The dogs were also classified as high or low for each nutrient category (protein, fat, and crude fiber) based on whether the nutrient was above or below the median value for each nutrient across the participants.

### 2.3. Sample Collection

Here, 5 mL of urine was collected via antepubic cystocentesis from each participant. Prior to collection, the skin at the collection site was thoroughly cleansed with 70% isopropyl alcohol. The collection needle was exchanged for a new, sterile needle prior to transfer of the urine to a sterile storage tube. Following urine collection, the samples were immediately refrigerated and then transported to a freezer (−80 °C) within 2 h. Mid-stream voided urine was also collected for each patient within 6 h following cystocentesis, and an aliquot ranging from 1 to 6 mL of the voided urine was submitted for a urinalysis and aerobic bacterial culture.

Dogs were excluded from further analyses if pyuria (defined as >5 white blood cells/hpf) or cytologic bacteriuria was present. The urine specific gravity (USG) was measured using a digital veterinary refractometer (MISCO Palm Abbe, Solon, OH, USA). The urine pH was determined using a urine dipstick chemistry test (Siemens MultiStix, Malvern, PA, USA). For the bacterial culture, approximately 0.5 mL of urine was gently mixed, followed by streak plate inoculation of a Blood Agar Plate (Hardy Diagnostics, Santa Maria, CA, USA) using a 1 µL inoculation loop (Globe Scientific, Mahwah, NJ, USA). The sample was incubated at 37 °C for 48 h and then assessed for colony growth. The samples with bacterial growth ≥10^5^ CFU/mL were excluded [32].

### 2.4. DNA Isolation and Amplicon Sequencing

Bacterial 16S rRNA gene amplification and sequencing was performed on each 5 mL urine sample. Each sample was centrifuged at 3000 rpm for 15 min to pellet the urine prior to additional processing [33]. The extraction of microbial DNA, library preparation, PCR amplification, and amplicon sequencing were performed at the University of Minnesota Genomics Center. The microbial DNA was extracted from each sample using the DNeasy PowerSoil Pro Kit (QIAGEN, Hilden, Germany). Two negative controls consisting only of DNA extraction reagents were included to assess for contaminants introduced during DNA isolation, library preparation, and PCR amplification. The extraction of microbial DNA from a commercially available mock bacterial community (ZymoBIOMICS Microbial Community Standard, Irvine, CA, USA) was performed as a positive control to evaluate the performance of the DNA isolation and sequencing methods. The DNA extractions and library preparation were performed in a single batch. The concentration and purity of the DNA were assessed using a Quanti-iT PicoGreen dsDNA Assay Kit (Invitrogen, Waltham, MA, USA) and NanoDrop spectrophotometry (Thermo Fisher Scientific, Waltham, MA, USA). The amplification of the V4 region of the bacterial 16S rRNA gene was performed using the primers 515F (GTGCCAGCMGCCGCGGTAA) and 806R (GGACTACHVGGGTWTCTAAT) [34], followed by amplicon sequencing using the MiSeq sequencing platform, with 2 × 300 base pair paired-end reads, as well as v3 chemistry (Illumina, San Diego, CA, USA). The raw sequence data are available at the National Center for Biotechnology Information BioProject PRJNA995758.

### 2.5. Processing of Raw DNA Sequence Reads

The primers were removed from raw, paired-end sequence reads using Cutadapt [35], followed by additional processing using QIIME2 (v 2020.8) [36] and DADA2 [37]. The outputs of high-resolution amplicon sequence variants (ASVs) were assigned taxonomic designations using the Silva reference database (v 138) [38]. Filtration of raw sequence data included the removal of chimeras, mitochondria, chloroplasts, ASVs with unassigned taxonomy, and ASVs present in only a single sample. Additionally, sequences were removed if represented in fewer than 10 total reads across all samples, as previously described [23].

Putative contaminants were identified and removed using the *decontam* package (v. 1.10.0) [39], based on the prevalence method and a threshold of 0.5. Using these criteria, ASVs were classified as a contaminant if identified in a larger proportion within negative controls than in clinical samples. After filtration and contaminant removal, a minimum of 300 sequence reads was required for inclusion in the downstream analyses, and any sample with fewer than 300 reads was removed from the dataset [29]. A relative abundance transformation was performed on all sequence read counts to normalize the data prior to the downstream analysis.

### 2.6. Statistical Analysis

The data analyses were performed using various packages within the R statistical software program. The normality of the continuous variables was analyzed using the Shapiro–Wilk test. The age and body weight demonstrated a normal distribution and are reported as mean values (±standard deviation, SD). The variables that did not exhibit a normal distribution (calories, protein, fat, and crude fiber) or ordinal variables (BCS) were reported as a median (range).

Alpha and beta diversity analyses were performed using the *vegan* (v. 2.5-7) [40] and *phyloseq* (v. 1.34.0) [41] packages. The alpha diversity (microbial diversity within groups) was calculated using the Shannon diversity index, inverse Simpson diversity index, and observed richness [42]. The alpha diversity values were compared between sex, diet groups (diet group 1 vs. 2), and dietary diversity groups (LDD versus HDD) using the Wilcoxon rank-sum test. Three measures of beta diversity (microbial diversity between groups) were calculated—Bray–Curtis dissimilarity, weighted UniFrac, and unweighted UniFrac [42]. The statistical differences in beta diversity between groups were determined using a permutational analysis of variance (PERMANOVA) with 1000 permutations. The differential abundance of specific taxa between groups was assessed using an indicator species analysis from the *labdsv* package (v. 2.0-1) [43,44], which assigns an indicator value (IV) to each ASV based on its relative frequency and abundance between groups. Taxa with an IV > 0.5 and a *p* value < 0.05 were considered differentially abundant, as previously described [23]. For taxa meeting these criteria, Wilcoxon rank-sum tests were also performed. The figures were generated using the *ggplot2* graphical imaging package (v. 3.3.6) [45].

## 3. Results

### 3.1. Study Participants

Nineteen dogs were recruited. One dog was excluded due to an owner-reported diet transition within one month of sample collection, and three dogs were excluded for insufficient sequence depth of urine samples (<300 reads). Therefore, the final study group consisted of 15 dogs, including 9 spayed females and 6 neutered males. The breeds included mixed (n = 6); Labrador Retriever (n = 3); and one each of Beagle, Cavalier King Charles Spaniel, Doberman Pinscher, English Bulldog, Miniature Goldendoodle, and Standard Poodle. Two of the 15 dogs (one male and one female) were from the same household. Additional participant and sample metadata are summarized in Table 1.

No dogs had glucosuria, ketonuria, or gross hematuria, although nine dogs exhibited microscopic hematuria in the urinalysis. One urine sample showed scant bacterial growth, below the level of exclusion, on the aerobic culture (<10^5^ CFU/mL). The remaining samples exhibited no observable bacterial growth. Nine dogs were not receiving any systemic medications aside from routine flea, tick, and heartworm preventatives. One dog had a history of well-controlled hypothyroidism and was receiving L-thyroxine. Two dogs received nutraceutical joint supplements, and three dogs received medications for anxiety only when needed (trazodone, n = 2; cannabidiol, n = 1).

### 3.2. Dietary Histories

All dogs consumed diets commercially formulated to meet maintenance nutritional needs. Eleven total diets were represented across the 15 dogs; 8 diets were consumed by a single participant, 2 diets were consumed by 2 dogs each, and 1 diet was consumed by 3 dogs (Table S1). One dog routinely consumed a daily mixture of two diets; one of these diets represented 60% of the dog’s daily caloric intake and was classified as the primary diet. Of the 11 diets represented across participants, 7 were produced by the same commercial brand (Purina^®^, Nestlé Purina Petcare, St. Louis, MO, USA), including each of the 3 diets consumed by more than 1 participant. Therefore, 11 dogs were consuming diets from this commercial brand and were designated diet group 1. The remaining 4 dogs consumed diets from 3 distinct commercial brands (Royal Canin^®^ USA Inc., Mars Inc., Saint Charles, MO, USA; NutriSource^®^ Pet Foods, Perham, MN, USA; Fromm^®^ Family Pet Food, Mequon, WI, USA) and were classified as diet group 2. No dogs were consuming prescription urinary diets.

Six dogs consumed 3 or more unique diets or treat sources (HDD group: 2 with 3 food sources, 2 with 5 food sources, and 2 with an unknown quantity of mixed treat sources) and 9 dogs consumed less than 3 unique food sources (LDD group: 5 with 1 food source and 4 with 2 food sources). An overlap between the HDD group and diet group 2 was observed. Of the 4 dogs in diet group 2, 3 were also in the HDD group. For the two dogs from the same household, both were in diet group 1; one was in the HDD group and the other was in the LDD group. The dietary data across all study participants are summarized in Table 2 and Table S2.

### 3.3. Data Processing and Bacterial Composition of Urine Samples

**Figure 1:**
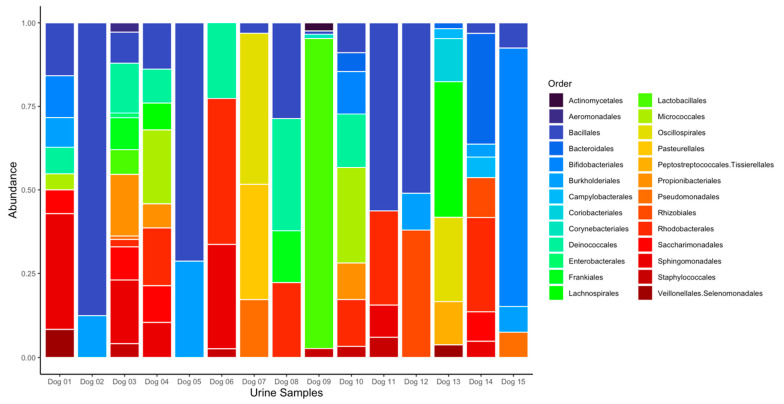
Bar plots of bacterial orders present in urine samples from 15 healthy dogs. Relative abundance of bacterial orders are shown from individual canine urine samples. Twenty-six orders were represented across all samples. Dog 7 and dog 12 were from the same household.

Eight sequences were identified as contaminants by the *decontam* package and were removed from the dataset prior to the downstream analysis (Table S3). After data filtering and contaminant removal, a total of 303,839 sequence reads were identified across the 15 urine samples (median 3373; range 337 to 134,198), represented by 76 unique ASVs. No sequence reads were present in the negative controls after filtering and contaminant removal. Each of the 8 organisms reported to be present in the commercial mock community was identified via 16S rRNA amplicon sequencing in the positive control, and no additional DNA sequences were identified from this sample. The most common phyla represented across samples were *Bacillota*, *Pseudomonadota*, and *Actinomycetota*, and the most common genera across all samples were *Streptococcus* (order *Lactobacillales*)*, Bifidobacterium* (order *Bifidobacteriales*), and *Anaerobacillus* (order *Bacillales*). The dominant taxa differed between the two dogs from the same household. A taxa bar plot of each urine sample reporting the relative abundance of bacteria at the order level is displayed in Figure 1.

### 3.4. Impact of Nutritional Features on Urobiome Composition

**Figure 2:**
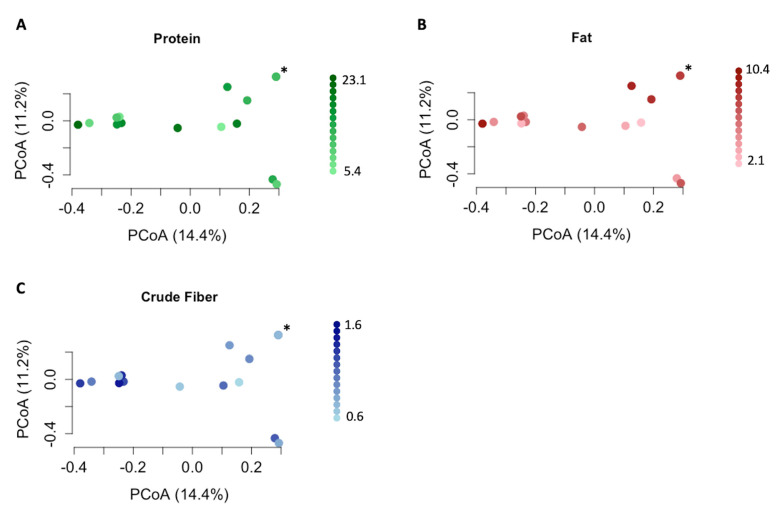
Bray–Curtis measures of beta diversity in 15 healthy canine urine samples, in association with protein, fat, and crude fiber contents. A principal coordinates analysis (PCoA) plot of beta diversity, as measured by the Bray–Curtis dissimilarity matrix, is presented for the three nutrients. Each point represents the urobiome of an individual dog. green, red, and blue represent the protein, fat, and crude fiber levels, respectively. Darker colors indicate higher respective nutrient intake and lighter colors indicate lower nutrient intake. Scales show the ranges of values for each nutrient (g/100 kcal): (**A**) protein (*p* = 0.73, R^2^ = 0.066); (**B**) fat (*p* = 0.88, R^2^ = 0.061); (**C**) crude fiber (*p* = 0.11, R^2^ = 0.084). Two points representing the urobiome from two separate female dogs are overlapping and are indicated by an asterisk (*).

**Figure 3:**
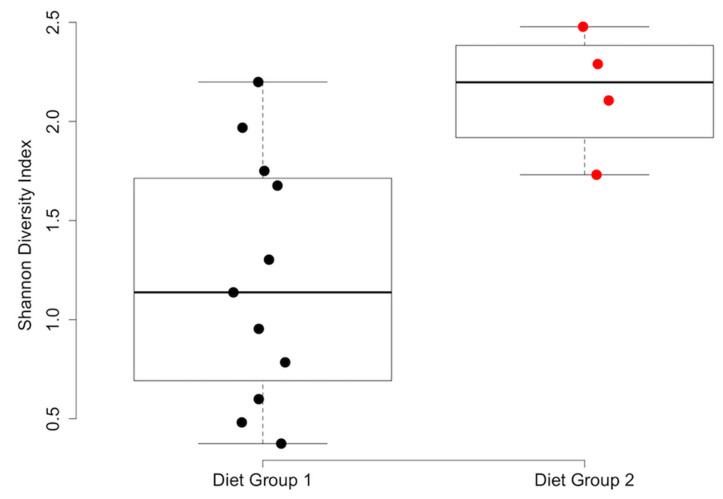
Shannon index values of alpha diversity by diet group. Box plot of the Shannon diversity index measure of alpha diversity is reported for the urine samples of dogs in diet group 1 (black circles) and diet group 2 (red circles). A Wilcoxon rank-sum test of alpha diversity was calculated between groups, *p* = 0.018. The boxes represent the 25th and 75th percentiles. Whiskers represent 1.5 times the interquartile range.

**Figure 4:**
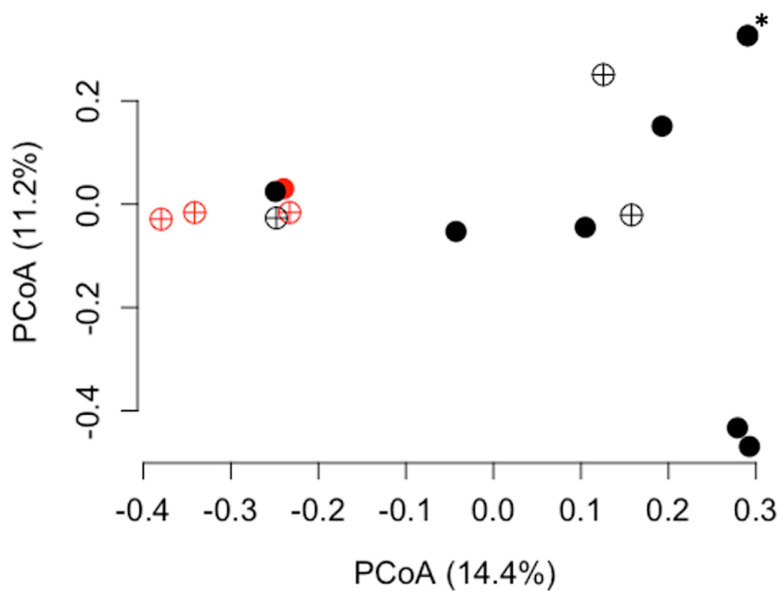
Bray–Curtis beta diversity values by diet group and dietary diversity. A principal coordinates analysis (PCoA) plot of beta diversity, as measured by the Bray–Curtis dissimilarity matrix, is presented for diet group and dietary diversity. Each point represents the urobiome of an individual dog. Black points indicate diet group 1 and red points indicate diet group 2. Closed circles indicate the LDD group and cross-hatched circles indicate the HDD group. A PERMANOVA was performed by diet group (*p *= 0.017, R^2^ = 0.10) and by dietary diversity group (*p *= 0.019, R^2^ = 0.10). Two points representing the urobiome from two separate female dogs are overlapping and are indicated by an asterisk (*). LDD = low dietary diversity; HDD = high dietary diversity.

**Figure 5:**
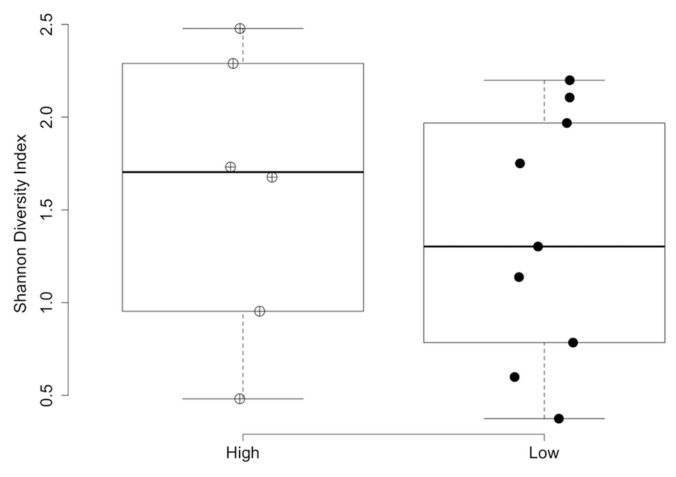
Shannon index of alpha diversity by dietary diversity group. A box plot of the Shannon diversity index measure of alpha diversity is reported for the urine samples of dogs in the HDD group (cross-hatched circles) and the LDD group (closed circles). A Wilcoxon rank-sum test of alpha diversity was calculated between groups, *p *= 0.61. The boxes represent the 25th and 75th percentiles. Whiskers represent 1.5 times the interquartile range. HDD = high dietary diversity; LDD = low dietary diversity.

The results of the statistical analyses of alpha and beta diversity across the comparison groups are summarized in Table 3. No differences in alpha or beta diversity (Figure 2) were detected in relation to the three nutrient profiles. However, multiple differences were observed between the diet groups. The dogs in diet group 1 exhibited significantly lower alpha diversity than those in diet group 2 according to the Shannon (*p* = 0.018; Figure 3) and inverse Simpson indices of diversity (*p* = 0.026). The beta diversity based on Bray–Curtis distances also differed between the dogs in diet group 1 compared to those in diet group 2 (R^2^ = 0.10, *p* = 0.017, Figure 4). The dogs in the HDD group exhibited significant differences in Bray–Curtis dissimilarity (R^2^ = 0.10, *p* = 0.019, Figure 4) as compared to the LDD group. The alpha diversity measures (Figure 5) did not significantly differ between the dietary diversity groups. No statistical differences in any alpha or beta diversity metrics were observed between the male and female study participants.

The differential abundance testing between the dogs in diet group 1 versus diet group 2 revealed that the dogs in diet group 1 harbored lower relative abundance levels of *Staphylococcus* (IV = 0.75, *p* for IV < 0.01, *p* for Wilcoxon rank-sum test < 0.01), *Bacillus halodurans* (IV = 0.67, *p* for IV = 0.036, *p* for Wilcoxon rank-sum test = 0.062), and *Paracoccus* (IV = 0.60, *p* for IV = 0.027, *p* for Wilcoxon rank-sum test = 0.036). *Anaerobacillus* was enriched in the HDD group (IV = 0.97, *p* for IV < 0.01, *p* for Wilcoxon rank-sum test < 0.01) compared to the LDD group.

## 4. Discussion

The aim of this pilot study was to identify associations between nutrient intake and urobiome composition in healthy adult dogs. The dietary intake levels of protein, fat, or crude fiber did not explain the bacterial composition of the urine samples in this study. While the taxonomic profiles of the canine gut microbiome are affected by diet, large variations in macronutrient profiles are typically required to produce observable taxonomic changes [46]. In the current study, a relatively narrow range of nutrient intake profiles for the primary diets was present across the study participants, which could explain the lack of urobiome effects based on the overall nutrient profiles. Furthermore, nutrient profiles alone do not capture all dietary information that may influence microbial communities and their impact on host health [47,48]. Microbial shifts secondary to diet might be more affected by nutrient subcategories (e.g., fiber types), specific ingredients, or micronutrient profiles than macronutrient intake alone. A more diverse spectrum of diets, represented by a broader range of nutrient profiles and commercial diet brands, might improve the ability to detect microbial shifts secondary to diet.

Dogs consuming a single brand of commercial dog food (diet group 1) exhibited lower alpha diversity scores and differences in the Bray–Curtis measure of beta diversity as compared to dogs consuming any other diet brand fed to dogs in this study. Three organisms (*Staphylococcus*, *Bacillus halodurans*, and *Paracoccus*) were lower in abundance in the urine samples of the dogs in diet group 1. Each of these genera have been previously identified as components of the urobiome in dogs [23,24], although the biological significance of their variation in abundance by diet brand remains unclear. A proposed explanation for the differences between diet groups is that urobiome shifts secondary to commercial diet brand result from differences in ingredient sourcing and processing between diet companies. For instance, dietary ingredients can influence the urine pH and the gut microbiome, particularly individual fiber sources [19,49]. By pairing microbiome analysis of urine with that of both stool samples and of food sources themselves, the origin of specific microbes could be identified, allowing better definition of the diet-gut-urinary microbiome axis. Importantly, this study was not designed to determine how specific diets relate to urobiome health, and the clinical relevance of urobiome differences between diet brands is unknown.

The Bray–Curtis measures of beta diversity and urine microbial profiles also differed based on the extent of dietary diversity. One organism (*Anaerobacillus*) was over-represented in the urine samples of the dogs consuming more diverse diets. To the authors’ knowledge, this organism has not been previously associated with dietary diversity in gut microbiome studies and is of unknown significance in the canine urobiome. In humans, increased dietary diversity positively correlates with gut microbiome stability [47] and diversity [50], although the relationship between dietary and microbiome diversity levels has been inconsistent across studies [47]. When evaluating the effect of dietary diversity on the gut microbiome or urobiome, the definition used for dietary diversity is important to consider. Various definitions have been proposed, ranging from simple counts of unique food items to more complex indices incorporating categorical food groups and relative proportions of consumed foods [31]. Given that all dogs in this study consumed a balanced commercial primary diet, dietary diversity was defined as the number of unique food sources consumed daily. We did not test whether using a different definition would change the results.

Importantly, differences in beta diversity based on both the diet and dietary diversity groups were only observed by the Bray–Curtis dissimilarity test and not by the weighted or unweighted UniFrac tests. Overall, this suggests that these factors produced minimal shifts in the abundance of phylogenetically related organisms. This finding also highlights the importance of testing multiple measures of beta diversity to assess for different patterns of microbial diversity.

This pilot study is limited by the high representation of a single commercial diet company and the small sample size overall, reducing the ability to detect smaller effects of nutrients or other dietary components on the urobiome. Additionally, the numbers of dogs in each diet and dietary diversity group were particularly small, and there was substantial overlap between these groups, with 3 of 4 dogs in diet group 2 also in the HDD group. Thus, we cannot definitively conclude whether one or both of these variables was truly driving the variations in urobiome composition. Household and environmental factors also contribute to the microbiome’s composition [12,51], and two dogs were from the same household in this study. Both dogs were in diet group 1 but they were in different dietary diversity groups. Although the impact of cohabitation on the canine urobiome is not fully understood, these dogs exhibited distinct urobiome taxonomic profiles despite a shared home environment (Figure 1). Additionally, both menstruation and menopausal status influence the urobiome’s composition in women, although the effects of spay and neuter status on the urobiome of dogs remains incompletely understood [52,53]. Therefore, sexually intact animals were excluded from the study given the potential confounding variables related to reproductive hormones on urobiome composition in dogs. Future studies evaluating the impact of the reproductive status on the canine urobiome’s composition and the effects of diet on the urobiome of sexually intact animals are warranted.

Another limitation of the study is the characterization of the urobiome through short-amplicon sequencing of the bacterial 16S rRNA gene. While this method offers an effective and efficient method for taxonomically profiling microbial communities, it does not determine microbial viability, function, or biological significance factors. In human urine samples, more than 80% of microbial DNA sequences detected with 16S rRNA gene sequencing are culturable when using an expanded quantitative urine culture (EQUC) [5]. Standard urine cultures are primarily designed to detect uropathogens and lack sensitivity for detecting most commensal urinary microbes, whereas the EQUC method uses more diverse growth conditions for enhanced culture sensitivity [5]. In future studies, we could gain more insight into microbial viability by pairing DNA sequencing with EQUC testing. Additionally, expanding this study to include shotgun metagenomics and metabolomics would offer greater insight into the functional responses of the urobiome to different diet brands, dietary diversity, or other dietary features. The other limitations pertained to our ability to extract and analyze nutrient data. Two dogs had known treat amounts from a select group of treats, although the precise number of each individual treat type was unknown. Some study participants received medications or supplements with unknown effects on urobiome composition. Additionally, several food sources, particularly treats, only had guaranteed analyses available, creating limitations when extracting comprehensive nutrient data. For instance, analyses were performed using crude fiber rather than total dietary fiber due to the inconsistent availability of total dietary fiber measurements. The total dietary fiber is the preferred measure for comparing fiber across diets, as crude fiber does not incorporate soluble fiber and does not reliably reflect the total dietary fiber [54]. This study was also unable to incorporate the relative digestibility levels of different food sources into the analyses. For human food sources, the U.S. Department of Agriculture Nutrient Database was used to obtain details about calorie and nutrient contents, as recommended in current veterinary nutrition guidelines [30].

## 5. Conclusions

This pilot study provides important foundational evidence that dietary features might impact the urobiome’s composition in healthy adult dogs. Specifically, both the type of commercial diet brand and dietary diversity correlated with the urobiome’s structure and diversity, although associations between protein, fat, or crude fiber and the urobiome were not observed in this small study group. Given the emerging evidence that the urobiome affects urinary tract health, the relationship between diet and urinary microbes has potential clinical relevance and could lead to the development of diet-based, clinical interventions for urobiome health. Moving forward, dietary data should be collected and incorporated into urobiome analyses when possible. Prospective, controlled, cross-over feeding trials are recommended to better define the relationships between the diet and urobiome.

## Figures and Tables

**Table 1:** Summary of participant metadata from 15 healthy dogs.

Variable	Value
Age (years)	3.7 (±1.8)
Weight (kg)	19.3 (±11.2)
BCS (scale 1–9)	5.5 (4.5–6.5)
Sex	FS (9), MN (6)
USG	1.042 (1.006–1.045)
Urine pH	7.3 (±0.82)

Normally distributed data are displayed as the mean ± standard deviation, while ordinal data or those that did not follow a normal distribution are reported as the median (range). BCS = body condition score; FS = female spayed; MN = male neutered; USG = urine specific gravity.

**Table 2:** Summary of dietary data from 15 healthy dogs.

Variable	Value
% of RER consumed	123 ± 49.2 ^1^
Kcal from treats (%)	7.5 ± 13.2 ^2^
Protein (g/100 kcal)	7.1 (5.4–23.1)
Fat (g/100 kcal)	3.5 (2.1–10.4)
Crude fiber (g/100 kcal)	1.1 (0.6–2.7)
Dogs in Diet Group 1	11
Dogs in Diet Group 2	4
Unique diet and treat sources	2 (1–5) ^3^
Dogs in HDD Group (≥3)	6
Dogs in LDD Group (<3)	9

Normally distributed data are displayed as the mean ± standard deviation and data that did not follow a normal distribution are reported as the median (range). The reported calories and nutrient values include the sum from the respective nutrient across all reported food sources, including both primary diet and treats. HDD = high dietary diversity; LDD = low dietary diversity; RER = resting energy requirement. ^1^ RER was calculated using the following formula: RER (kcal/day) = (ideal body weight kg ^3/4^) × 70. ^2^ Two dogs consumed mixed treats from a commercial treat delivery service. The kcal totals of treats reported here are based on the average calorie content from all treats. ^3^ The exact number of unique treats consumed by these two dogs was unknown and may have exceed this reported range.

**Table 3:** Summary of alpha and beta diversity values between comparison groups.

Diversity Metric	Protein	Fat	Crude Fiber	Diet Groups	Dietary Diversity Groups
Alpha Diversity (*p* value)					
Shannon	1	0.23	0.39	0.018	0.61
Inverse Simpson	0.87	0.34	0.28	0.026	0.61
Observed Richness	0.82	0.56	0.91	0.077	0.26
Beta Diversity (*p* value, R^2^)					
Bray–Curtis	0.73, 0.066	0.88, 0.061	0.11, 0.084	0.017, 0.10	0.019, 0.10
WUF	0.46, 0.067	0.46, 0.070	0.50, 0.067	0.13, 0.090	0.51, 0.070
UUF	0.12, 0.091	0.31, 0.080	0.63, 0.064	0.062, 0.11	0.19, 0.090

The *p* values for Wilcoxon rank-sum tests of three measures of alpha diversity are reported for each comparison group—high- and low-nutrient categories (protein, fat, and crude fiber), diet group (diet group 1 and diet group 2), and dietary diversity group (HDD and LDD). The *p* values and R^2^ values based on the PERMANOVA for each measure of beta diversity are reported. For beta diversity, statistical testing using a PERMANOVA was performed using continuous nutrient values. WUF = weighted UniFrac; UUF = unweighted UniFrac; HDD = high dietary diversity; LDD = low dietary diversity.

## Data Availability

The raw sequence data generated by and analyzed within this study are available through the National Center for Biotechnology Information (NCBI) Sequence Read Archive (SRA) under the BioProject number PRJNA995758. Other data are included in the manuscript or additional files.

## Supplementary Materials

The following supporting information can be downloaded at https://www.mdpi.com/article/10.3390/vetsci11070286/s1: Table S1. Diet brands and formulations consumed by 15 healthy dogs. Table S2. Nutrient profiles consumed daily be each individual dog. Table S3. Putative contaminant sequences identified via *decontam* from canine urine samples.
