# Supplementary material for: Dietary Features Are Associated with Differences in the Urinary Microbiome in Clinically Healthy Adult Dogs
Source: Vet Sci. 2024 Jun 27;11(7):286. doi: 10.3390/vetsci11070286 (PMC11281549; doi:10.3390/vetsci11070286)
Supplement: Supplementary file 1 [file vetsci-11-00286-s001.zip › S1_Table.pdf]

Table S1: Diet brands and formulations consumed by 15 healthy dogs

**Diet**	**Number of Dogs**
Purina® Pro Plan® Adult Complete Essentials Shredded Blend Chicken & Rice Dry Dog Food	3
Purina® Pro Plan® Adult Large Breed Shredded Blend Chicken & Rice Formula Dry Dog Food	2
Purina® Pro Plan® Adult Small Breed Chicken & Rice Formula Dry Dog Food	2
Purina® Beneful® Healthy Weight Dry Dog Food with Farm-Raised Chicken	1
Purina® Dog Chow® Complete Adult Chicken Flavor Dry Dog Food	1
Purina® ONE® Lamb & Rice Formula Dry Dog Food	1
Purina® Pro Plan® Veterinary Diets EN Gastroenteric® Canine Formula	1
Fromm® Four-Star Nutritionals® Duck À La Veg® Recipe	1
NutriSource® Beef & Rice Recipe	1
Royal Canin® Bulldog Adult Dry Dog Food	1
Royal Canin® Labrador Retriever Puppy Dry Dog Food	1
