# Supplementary material for: Dietary Features Are Associated with Differences in the Urinary Microbiome in Clinically Healthy Adult Dogs
Source: Vet Sci. 2024 Jun 27;11(7):286. doi: 10.3390/vetsci11070286 (PMC11281549; doi:10.3390/vetsci11070286)
Supplement: Supplementary file 1 [file vetsci-11-00286-s001.zip › S2_Table.pdf]

Table S2: Nutrient profiles consumed daily by each individual dog

Dog ID	Body weight (kg)	BCS (1-9 scale)	Daily Caloric Intake (kcals)	Protein (g/100 kcal)	Fat (g/100 kcal)	Fiber (g/100 kcal)
1	9.1	5	533	10.6	3.58	0.63
2	43	6.5	1059	7.05	3.25	1.22
3	23.6	5	1081	8.94	3.49	1.3
4	19	6	1085	23.09	10.36	1.39
5	11.4	5	587	6.41	3.94	0.75
6	29.5	6.5	568	5.41	2.57	1.16
7	26	4.5	883	7.05	3.25	1.22
8	26.4	5.5	577	8.79	2.38	2.7
9	10	6.5	757	9.2	2.06	0.57
10	9.1	5.5	393	5.72	3.38	1.56
11	22.7	6	696	6.13	3.33	1.14
12	4.1	4.5	452	7.34	4.28	0.77
13	4.5	5.5	204	6.36	3.84	0.76
14	31.8	6	789	6.39	3.93	0.75
15	18.8	5.5	362	6.86	4.22	0.79
