# Supplementary material for: Dietary Features Are Associated with Differences in the Urinary Microbiome in Clinically Healthy Adult Dogs
Source: Vet Sci. 2024 Jun 27;11(7):286. doi: 10.3390/vetsci11070286 (PMC11281549; doi:10.3390/vetsci11070286)
Supplement: Supplementary file 1 [file vetsci-11-00286-s001.zip › S3_Table.pdf]

Table S3. Putative contaminant sequences identified via *decontam* from canine urine samples

**Organism**	**P**
Escherichia-Shigella	.17
Ruminococcus gnavus group	.06
Paracoccus.1	.18
Anaerobacillus	.19
Paracoccus.2	.17
Cutibacterium	.14
Burkholderia-Caballeronia-Paraburkholderia	.08
Rhodobacteraceae.1	.04

P is a composite score statistic that is assigned to each amplicon sequence variant (ASV). Using a threshold of 0.5 and the prevalence method, features are classified as contaminants if present in a higher fraction of negative controls than in biological samples [38]. Each ASV classified as a contaminant was removed from the dataset.
